# Association between fertility treatments and breast cancer risk in women with a family history or BRCA mutations: a systematic review and meta-analysis

**DOI:** 10.3389/fendo.2022.986477

**Published:** 2022-09-13

**Authors:** Xiaojing Liu, Jing Yue, Ruqiya Pervaiz, Hanwang Zhang, Lan Wang

**Affiliations:** ^1^ Reproductive Medicine Center, Tongji Hospital, Tongji Medical College of Huazhong University of Science and Technology, Wuhan, China; ^2^ Faculty of Chemical and Life Science, Department of Zoology, Abdul Wali Khan University, Mardan, Pakistan

**Keywords:** breast cancer, brca mutation, infertility, ovarian stimulation, fertility treatment

## Abstract

Women with hereditary breast cancer factors are more likely to be infertile and tend to receive fertility treatments. The safety of fertility treatments that contain hormone-related medications for ovarian stimulation has gained wide attention; however, evidence of the safety of fertility treatments is limited. This study aims to assess the association between fertility treatments and the incidence rate of breast cancer in women with a family history of breast cancer or *BRCA* mutations. A literature search was conducted in PubMed, Cochrane Library, and Embase. Studies concerning the effect of fertility treatments on breast cancer risk in genetically susceptible women were included. The fixed and random effects models were used to estimate the summary effects. Risk Of Bias In Non-randomized Studies - of Interventions instrument was used to assess the risk of bias in the included studies. A total of 5,282 studies were screened. Five cohort studies and three case-control studies were included. Breast cancer risk was not significantly increased by fertility treatments in general genetically susceptible women [pooled odds ratio (OR) 1.18, 95% confidence interval (CI) 0.96–1.45], women with a family history of breast cancer (pooled OR 1.35, 95% CI 0.97–1.89), or women with BRCA mutations (pooled OR 1.02, 95% CI 0.74–1.4). In subgroup analyses, there was no significant increase in breast cancer risk whether in BRCA1 mutation carriers (pooled OR 1.18, 95% CI 0.81–1.72), BRCA2 mutation carriers (pooled OR 0.54, 95% CI 0.09–3.34), or in the women treated with *in vitro* fertilization (pooled OR 0.75, 95% CI 0.51–1.1), clomiphene citrate (pooled OR 1.07, 95% CI 0.78–1.45) or gonadotropins (pooled OR 1.32, 95% CI 0.8–2.18). This is the first meta-analysis concerning the impact of fertility treatments on breast cancer risk in genetically susceptible women. Despite the finding that fertility treatment did not significantly increase breast cancer risk in genetically susceptible women, large prospective cohorts with more detailed information are required. Further investigations are needed to explore subtypes of breast cancer, genetic background of hormone-related breast cancer, and the association between BRCA mutations and the incidence of hormone receptor-positive breast cancer.

**Registration number:**

PROSPERO(CRD42021281336).

## Introduction

Breast cancer is the most common malignancy in female adults worldwide and is a leading cause of cancer-related deaths (1). Women who receive hormone therapy are considered to be at higher risk of breast cancer because hormone stimulation drugs could activate gonadotropins releasing and signaling, increase estrogen levels, potentially activate the steroid receptor-related oncogenic pathway and promote tumor progression (2–4).

The fertility medications include CC (clomiphene citrate), gonadotropins and letrozole. CC is a selective estrogen receptor modulator and could inhibit the negative feedback of gonadotropin releasing, thus promoting estrogen production and inducing ovulation (5). Gonadotropins include FSH (follicular stimulating hormone), LH (luteinizing hormone) and hCG (human chorionic gonadotropin), which bind to the receptor of ovarian follicular cells directly and initiate ovarian stimulation (5). However, the effect of hCG on breast cancer risk is controversial. Placental hCG is a candidate hormone with antitumoral effect in pregnancy, while the ectopic hCG promotes tumor progression (6). Letrozole is an aromatase inhibitor which could inhibit the negative feedback on FSH by preventing estrogen production and inducing ovulation (5). Letrozole is also used as a first-line therapy drug for hormone receptor positive breast cancers (7). However, the effects of combination of letrozole with other fertility medications on breast cancer risk remain unknown. With the increasing prevalence of female reproductive disorders, infertility is becoming a public health issue. As a result, the safety of ovarian stimulation drugs, which are the most prescribed hormone-related medications for fertility treatment, has received increasing attention.

A history of breast cancer in at least one first- or second- or third-degree relative and germline *BRCA1* or *BRCA2* mutations are also important risk factors for breast cancer in an inherited way (8, 9). Emerging evidence suggests a negative impact of *BRCA* mutations on ovarian reserve in women who present with lower serum anti-Müellerian hormone (AMH) (10, 11). Deleterious *BRCA* mutations, mostly *BRCA1*, were able to interrupt the process of DNA double strands repairing, causing damage to oocytes (12). Due to diminished ovarian reserve, *BRCA* mutation carriers are more likely to be infertile and may be required to receive fertility treatments. This causes a wide concern among genetically susceptible women regarding whether they are more likely to be diagnosed with breast cancer after receiving fertility treatments.

Despite multiple clinical studies and meta-analyses focused on the association between fertility treatments and breast cancer risk, and they have confirmed the safety of fertility treatments among general female population, there was no meta-analysis concerning the impact of fertility treatments on women with hereditary breast cancer factors (13–18). Even though some studies have been conducted directly among genetically susceptible women and they have not identified the harmfulness of fertility treatments, too (19–21); genetic professionals remain unsure of the safety of fertility medications among genetically susceptible women (22), considering the non-uniform follow-up years, complex regimens of fertility treatments, heterogeneity of the study population, and other confounding factors among the existing studies. To comprehensively evaluate the safety of fertility treatments among genetically susceptible women, a study is required to gather all related studies and systematically synthesize useful data from the existing studies. 

Therefore, to investigate whether fertility treatments increase the risk of breast cancer in women with hereditary risk factors, we reviewed the current literature, including randomized controlled trials (RCTs), case-control and cohort studies, to assess the association between fertility treatments and the incidence rate of breast cancer in women with a family history of breast cancer or *BRCA* mutations.

## Materials and methods

This study was conducted in accordance with the Meta-analyses of Observational Studies in Epidemiology (MOOSE) and Preferred Reporting Items for Systematic Reviews and Meta-analysis (PRISMA) 2020 guidelines (23, 24). The protocol containing the inclusion criteria and methods of analysis was registered at PROSPERO(CRD42021281336).

### Study eligibility

Randomized controlled, cohort and case-control studies focusing on the association between breast cancer incidence rates and fertility treatments were considered. Women with a family history of breast cancer or *BRCA* mutations (genetically susceptible women) who received fertility treatment were included in the study population. Fertility treatments encompassed assisted reproductive technology (ART), *in vitro* fertilization (IVF) or intracytoplasmic sperm injection (ICSI), and the use of ovarian stimulating drugs, such as CC, letrozole, and gonadotropins. The reference population included genetically susceptible women who did not receive fertility treatment. The endpoint was breast cancer diagnosis. There were no limitations on publication year or status. Case series or reports that enrolled fewer than 10 patients, studies with incomplete data or overlapping cohorts, and non-English language literature were excluded. Studies on patients diagnosed with breast cancer before receiving fertility treatments such as embryo or oocyte cryopreservation, were also excluded.

### Search strategy and study selection

Three electronic databases including PubMed, Cochrane Library, and Embase, were searched until September 1, 2021, to identify relevant studies. The MESH terms used in the search are as follows: (“Reproductive Techniques, Assisted” OR “Fertility Agents” OR “Infertility”) AND “Breast Neoplasm.” A detailed search strategy is presented in **Supplementary Table SI**.

The titles and abstracts of the included studies were browsed by two independent reviewers (W.L. and L.X.). Duplicates were excluded in addition to studies that were inconsistent with the eligibility criteria mentioned above. After the initial screening, the full texts of potentially relevant studies were inspected and assessed for eligibility. We sent emails to six authors for additional information. We received responses from two authors, and one of the responders could not provide further information because our request was unmatched by her data. The other four did not respond, perhaps because the system was invalid or they were unreachable. Discrepancies were resolved by discussion between the two authors to reach a consensus.

### Data extraction

We generated an Excel worksheet with a frame of key information for analysis. Information was extracted as follows: general information of the study (title, author, year, journal, country), study characteristics (study design, number of participants, mean age, exposure such as fertility-treatment protocol, type and dose of drugs, duration of follow-up), and outcomes including odds ratio (OR), hazard ratio (HR), relative risk (RR), and raw data for further calculation. Two reviewers (W.L. and L.X.) independently collected data from the selected articles and resolved disagreements through discussion.

### Data synthesis

The OR was used as a summary statistic for ORs, HRs, and RRs because the incidence rate was low. I² statistics with 95% confidence interval (CI) were used to evaluate the heterogeneity between studies. The result was classified as low heterogeneity when I^2^ was < 50%, moderate heterogeneity when I^2^ was ≥ 50% and < 75%, or high heterogeneity when I^2^ was ≥ 75%. Fixed-effects (inverse-variant) models were used to determine the summary estimate effects in the cases with low heterogeneity; random-effects (DerSimonian-Laird) models were used in the cases with moderate or high heterogeneity.

Subgroup analyses were conducted according to *BRCA* mutation types, including *BRCA1* and *BRCA2*, or types of fertility treatments, such as IVF, CC, or gonadotropins. Sensitivity analysis was conducted to assess whether the pooled estimates were stable. Stata 16.0 software (STATA Corporation, College Station, TX, USA) was used for the statistical analysis.

### Risk of bias and quality assessment

Risk Of Bias In Non-randomized Studies - of Interventions (ROBINS-I) instrument was used to assess the risk of bias in cohort studies and case-control studies by two independent reviewers (W.L. and L.X.) (25). Disagreements were resolved by discussion to reach a consensus. *Robvis* was used to create a risk-of-bias plot (26) (**Supplementary Fig. S1**).

## Results

### Study selection

**Figure 1:**
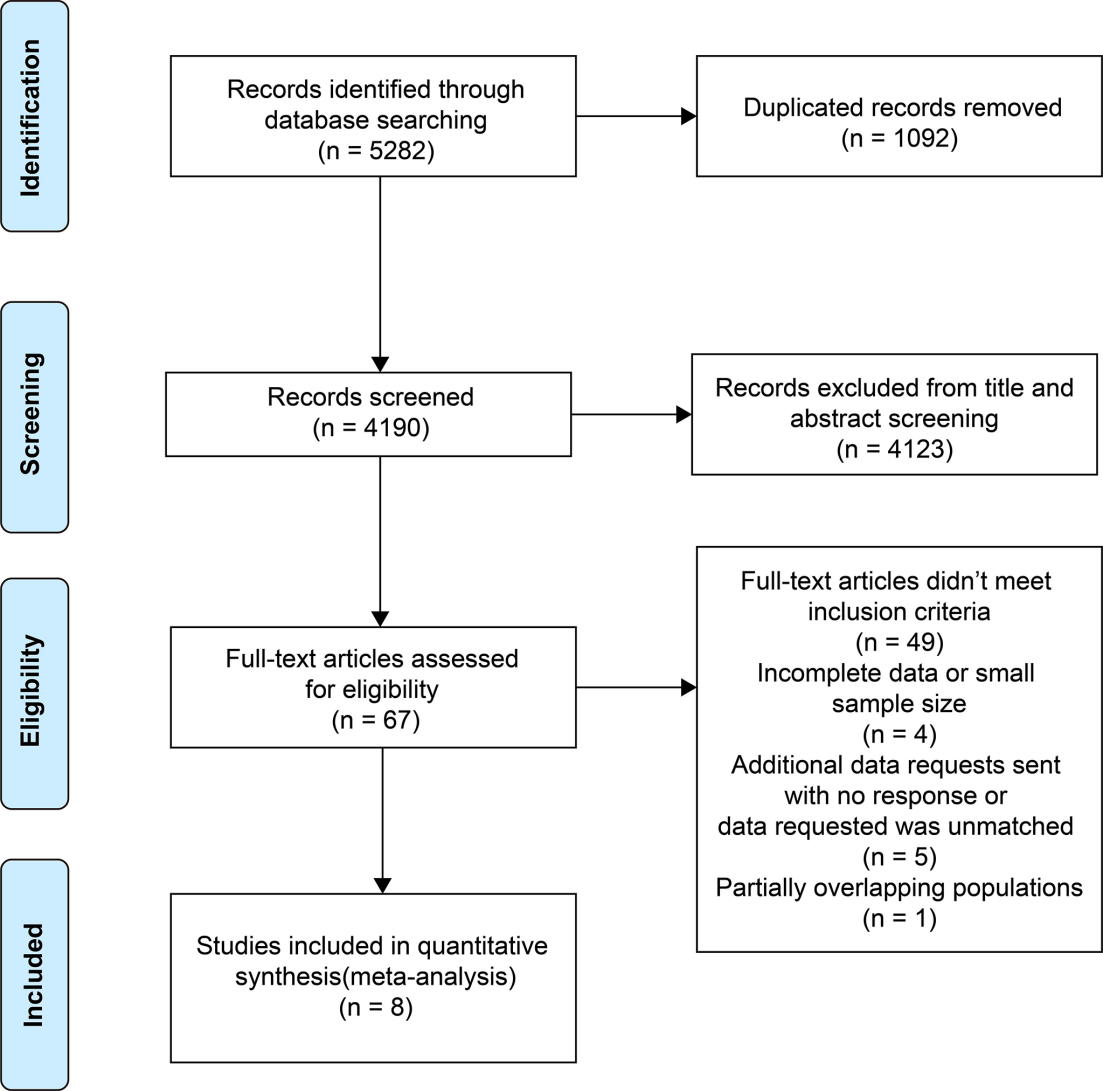
Preferred Reporting Items for Systematic Reviews and Meta-analysis (PRISMA) flow diagram of the study selection process.

A total of 5,282 studies were identified from the three electronic databases and 1,092 duplicated articles were excluded. After screening the titles and abstracts, 67 studies were selected for full-text viewing. 49 studies did not meet the inclusion criteria, and four studies were excluded because of incomplete data or small sample sizes. We requested additional information of six studies and received one positive reply. One study with an overlapping cohort population was excluded. One study (18) was included by the reviewers after a literature search was conducted on September 1, 2021. Finally, eight studies were included (20, 21, 27–32) (**Figure 1**).

### Study characteristics

The characteristics of the included studies are listed in **Table 1**. Among the eight studies published between 1996 and 2021, three were case-control studies (28, 29, 31), two were prospective cohort studies (20, 30) and three were retrospective cohort studies (21, 27, 32). A total of 4,352 genetically susceptible patients with breast cancer were included, of whom 350 women were treated with fertility treatments.

**Table 1:** Characteristics of included studies in the meta-analysis of the impact of fertility treatments on breast cancer risk in genetically susceptible women.

Study publication	Country	Study period	Study types	Types of hereditary factors	Cohort size (genetically susceptible women)	Total number of exposed women	Number of incidence cases	Number of exposed cases	Fertility treatments strategy
Perri et al. (2021)	Israel	1995-2020	Historical prospect cohort study	BRCA1/2 mutations	1,824	332	687	89	CC, gonadotropins, IVF and combination of these treatments
Derks-Smeets et al. (2018)	The Netherlands	2010-2013	Retrospective cohort study	BRCA1/2 mutations	2,514	76	938	15	Ovarian stimulation for IVF
Kotsopoulos et al. (2008)	Canada	1994-2007	Case-control study	BRCA1/2 mutations	2,760	137	1,380	70	IVF or fertility medication including CC, gonadotropins or other drugs
Braga et al. (1996)	Italy	1991-1994	Case-control study	Family history of breast cancer	434	12	299	2	Infertility treatments
Gauthier et al. (2004)	France	1990-2000	Prospective cohort study	Family history of breast cancer	10,221	526	455	32	Treated by fertility drugs including CC, menotrophin,and chorionic gonadotrophin
Pervaiz et al. (2018)	North Cyprus	2016-2017	Case-control study	Family history of breast cancer	358	77	228	59	Fertility drugs
Vassard et al. (2021)	Denmark	1994-2016	Retrospective cohort study	Family history of breast cancer	142,282	N.R.	289	36	ART
Brinton et al. (2014)	The USA	1965-2010	Retrospective cohort study	Family history of breast cancer	619	N.R.	76	47	CC, gonadotropins and combination of these treatments
**Study publication**	**Effect estimates (outcomes)**	**Reference group (matching controlling factors)**	**Adjusting factors**	**follow-up (years)**	**Ascertain of exposure**	**Ascertain of cancer**	**Risk of bias**	
Perri et al. (2021)	HR (breast cancer)	General population(mutation type, parental origin of the mutation, age at menarche, hormone replacement therapy, incidence of cancer diagnosis)	Risk-reducing bilateral salpingo-oophorectomy and/or prophylactic mastectomy, BRCA mutation type, parity, age at menarche, age at first pregnancy, OC use, paternal mutation origin	N.R.	Medical records	Israel National Cancer Registry	Moderate
Derks-Smeets et al. (2018)	HR (breast cancer)	General population or subfertile population (other invasive cancer diagnosis, bilateral prophylactic mastectomy, subfertility, birth cohort, other fertility treatments including clomid and/or intrauterine insemination, use of OC, parity, age at first birth)	Subfertility, birth year	N.R.	Medical record from the Dutch HEBON study (Hereditary Breast and Ovarian cancer study, the Netherlands) and the national PGD registry	Self-reported, the Dutch national Pathology Database (PALGA) and the Netherlands Cancer Registry (NCR)	Moderate
Kotsopoulos et al. (2008)	OR (breast cancer)	General population(mutation in the same gene, year of birth, country of residence and parity, diagnosis with other cancers, bilateral mactectomy)	Parity, age at mernache and ethnicity	N.R.	Medical records from 47 participating medical centres in nine countries	Medical records from 47 participating medical centres in nine countries	Moderate
Braga et al. (1996)	OR (breast cancer)	General population (age at inclusion, area of residence)	Menopausal status, parity, education, age, centre	N.R.	Medical records from the major teaching and general hospitals of study areas	Medical records from the major teaching and general hospitals of study areas	Serious
Gauthier et al. (2004)	RR (breast cancer)	General population (N.R.)	Parity and age at first full-term pregnancy, age at mernache, personal history of benign breast disease, number of first-degree relatives with a history of breast cancer, BMI at inclusion, active smoking at inclusion, numbers of years school	9.7	Medical records from the French E3N cohort(Etude Epidémiologique auprès de femmes de la Mutuelle Générale de l’Education Nationale)	Medical records from the French E3N cohort	Serious
Pervaiz et al. (2018)	OR (breast cancer)	General population (age)	N.R.	N.R.	Medical records from Near East Hospital and Dr. Burhan Nalbantoglu State Hospital	Medical records from Near East Hospital and Dr. Burhan Nalbantoglu State Hospital	Serious
Vassard et al. (2021)	HR (breast cancer)	General population (age)	Age(time-varying, stratified in 2-year intervals), education level, partnership status, year, nulliparity (time-varing)	9.69	IVF register	Danish Cancer registry	Serious
Brinton et al.(2014)	HR (breast cancer)	General population (exclusion of diagnosis within first year of fertility treatment)	Study site, calendar year of first infertility evaluation, gravidity at first clinic visit	30	Five reproductive enocrinology practices	Cancer registries in the 14 states	Serious

NR, not reported; HR, hazard ratio; RR, relative risk; OR, odds ratio; CC, clomiphene citrate; IVF, *in-vitro* fertilization; ART, assisted reproductive technology; OC, oral contraceptive; HMG, human menopausal gonadotropin; PGD, preimplantation genetic diagnosis.

Five studies reported women with breast cancer family history (21, 29–32), among which one study only described the independent effects of separate fertility drugs such as CC or gonadotropins (32) and four studies reported the synthetic effects of fertility treatments (21, 29–31). Three studies reported *BRCA* mutation carriers (20, 27, 28), among which two studies reported independent effects on women with *BRCA1* or *BRCA2* mutations (27, 28).

Three studies reported breast cancer risk in women who received IVF (20, 27, 28). Three studies reported breast cancer risk in women who received CC or gonadotropins separately (20, 28, 32).

Three studies excluded the impact of prophylactic mastectomy (20, 27, 28), and the other five studies did not mention whether they adjusted the surgical factor or not (21, 30–32).

Four studies demonstrated adjusted HR as an estimate effect (20, 21, 27, 32), three studies demonstrated adjusted OR as an estimate effect (28, 29, 31), and one study demonstrated adjusted RR as an estimate effect (30).

Three studies reported median or mean follow up duration ranging from 9.69 to 30 years (21, 30, 32).

### Fertility treatments and breast cancer risk in genetically susceptible women

**Figure 2:**
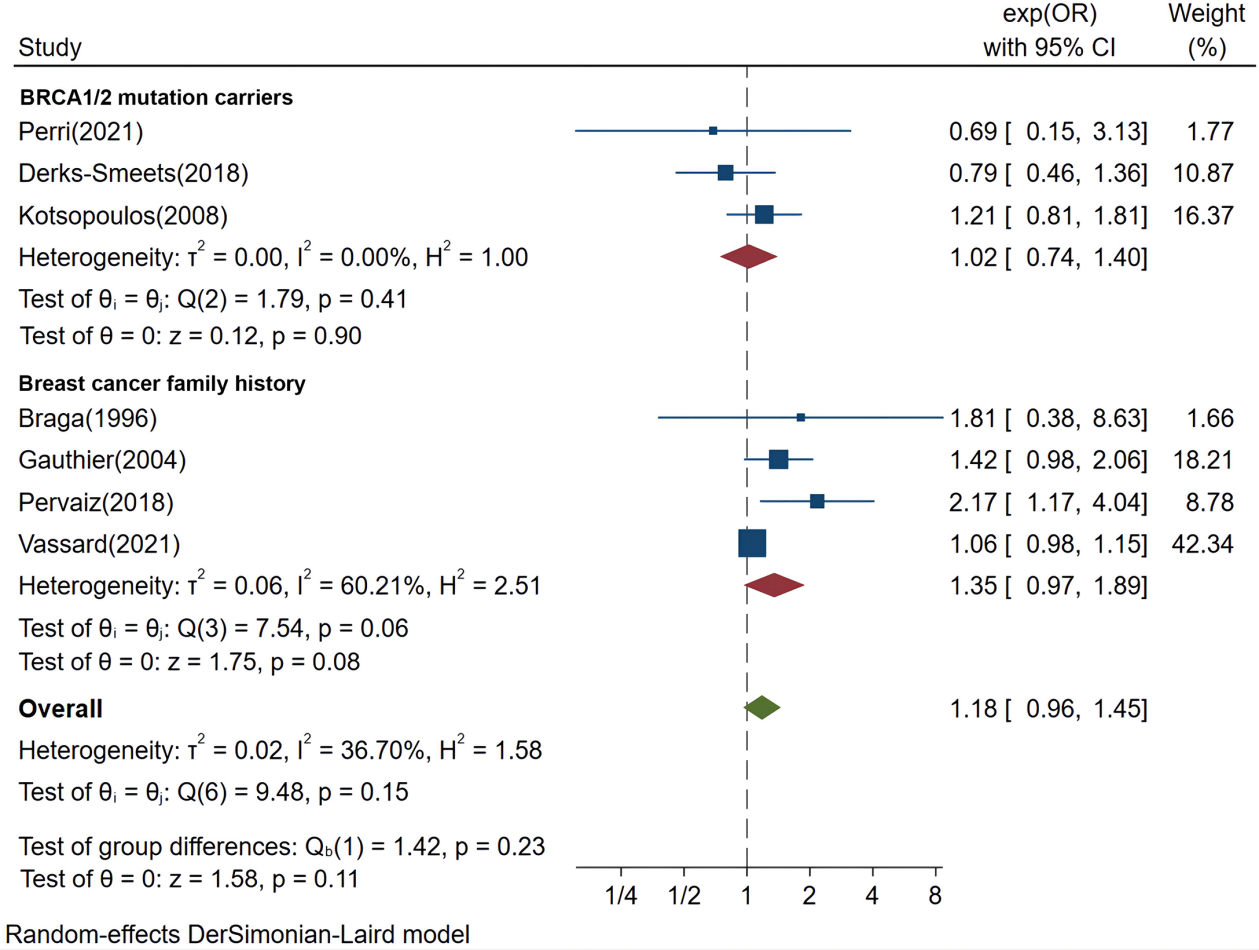
Forest plots demonstrating the comparison of breast cancer risk in women with hereditary factors treated with fertility treatments or not. OR, odds ratio.

Among the eight included studies, one study only described the independent effects of CC or gonadotropins (32); seven studies were included to compare synthetic effects of fertility treatments in general genetically susceptible populations (20, 21, 27–31), of which three studies reported *BRCA* mutation carriers (20, 27, 28) and four studies reported women with a family history of breast cancer (21, 29–31). A meta-analysis showed that there was no significant increase in breast cancer risk by fertility treatments in general genetically susceptible population (pooled OR 1.18, 95% CI 0.96–1.45, *P*=0.11, I^2 =^ 36.7%), in women with a family history (pooled OR 1.35, 95% CI 0.97–1.89, *P*=0.08, I^2 =^ 60.21%), or in women with *BRCA* mutations (pooled OR 1.02, 95% CI 0.74–1.4, *P*=0.9, I^2 =^ 0%) (**Figure 2**). Sensitivity analyses of the outcomes are shown in **Supplementary Tables SII-SIV**.

### Fertility treatments and breast cancer risk in *BRCA*1 or *BRCA*2 mutation carriers

**Figure 3:**
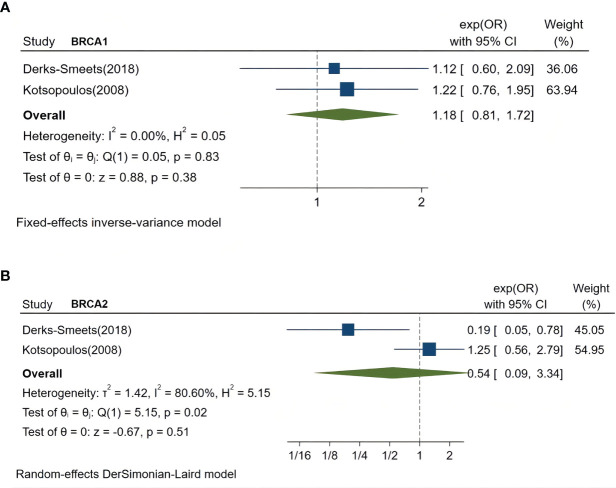
Forest plots demonstrating the comparison of breast cancer risk in women with *BRCA1* mutations or *BRCA2* mutations who were treated by fertility treatments or not. **(A)**, comparison of breast cancer risk in women with *BRCA1* mutations. **(B)**, comparison of breast cancer risk in women with *BRCA2* mutations. OR, odds ratio.

Two studies separately compared breast cancer risk in women with *BRCA1* or *BRCA2* mutations (27, 28). A meta-analysis showed no significant increase by fertility treatment in the breast cancer risk of *BRCA1* mutation carriers (pooled OR: 1.18, 95% CI 0.81–1.72, *P*=0.38, I^2 =^ 0%) (**Figure 3A**) or *BRCA2* mutation carriers (pooled OR 0.54, 95% CI 0.09–3.34, *P*=0.51, I^2 =^ 80.6%) (**Figure 3**).

### IVF or CC or gonadotropins and breast cancer risk in genetically susceptible women

**Figure 4:**
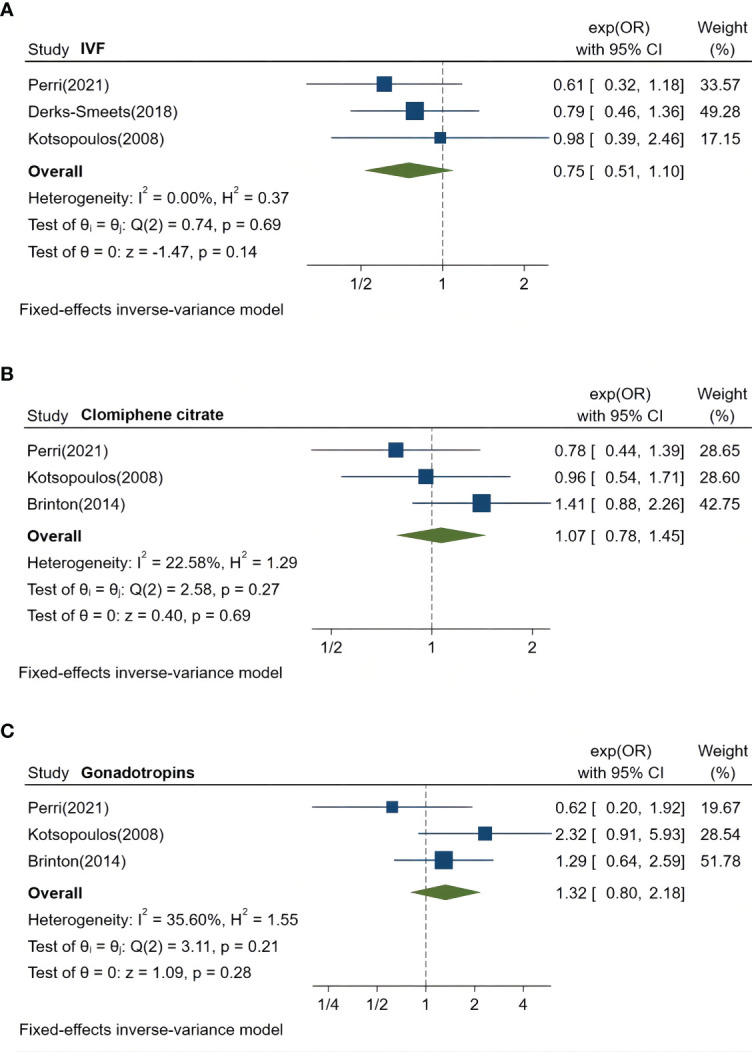
Forest plots demonstrating the comparison of breast cancer risk in genetically susceptible women who were treated with IVF, CC, or gonadotropins. **(A)**, comparison of breast cancer risk in genetically susceptible women treated by IVF or not. **(B)**, comparison of breast cancer risk in genetically susceptible women treated by CC or not. **(C)**, comparison of breast cancer risk in genetically susceptible women treated with gonadotropins or not. OR, odds ratio; CC, clomiphene citrate; *in vitro* fertilization, IVF.

Three studies compared breast cancer risk in women treated with IVF, and a meta-analysis showed no significant increase in breast cancer risk (pooled OR 0.75, 95% CI 0.51–1.1, *P*=0.14, I^2 =^ 0%) (**Figure 4**) (20, 27, 28). Three studies compared breast cancer risk in women treated with CC, and a meta-analysis showed no significant increase in breast cancer risk (pooled OR 1.07, 95% CI 0.78–1.45; *P*=0.69; I^2 =^ 22.58%) (**Figure 4**) (20, 28, 32). Three studies compared breast cancer risk in women treated with gonadotropins, and one meta-analysis showed no significant increase in breast cancer risk (pooled OR 1.32, 95% CI 0.8–2.18, *P*=0.28; I^2 =^ 35.6%) (**Figure 4C**) (20, 28, 32). Sensitivity analyses of the outcomes are shown in **Supplementary Tables SV-SVII**.

## Discussion

In this study, we observed that compared with population with similar genetic backgrounds who was not exposed to fertility treatments, the breast cancer incidence rate was not significantly increased by fertility treatments in women with a family history of breast cancer or *BRCA* mutations. No evident effects of fertility treatments on breast cancer risk were observed among women with different types of *BRCA* mutations or women treated with IVF, CC, or gonadotropins.

In our study, there was no evidence to support that fertility treatments have an impact on breast cancer incidence in genetically susceptible women; this was consistent with the findings of most previous meta-analyses on the general female population, in spite of some controversies among the previous studies that breast cancer risk under fertility treatments was lower compared with women who gave birth (14) and higher with non-IVF treatments or longer follow-up duration (33). On the contrary, no excess breast risk was found to be associated with more IVF cycles or follow-up years in Cullinane’s study (13). Considering the complexity of confounding factors and heterogeneity of population, this meta-analysis which first adjusted hereditary breast cancer factors is of great importance. Low to moderate heterogeneity in most outcomes and reasonable analysis methods demonstrated some degree of strength in the study.

Moderate heterogeneity (I^2 =^ 60.21%) was present in the five included studies concerning women with a family history of breast cancer, of which one study showed a notable increase in breast cancer risk by fertility treatments (OR 2.17, 95% CI 1.17–4.04) (31). In agreement with the finding from the sensitivity analysis of the family history group that OR was significantly increased after omitting another single study (OR 1.60, 95% CI 1.17–2.18) (**Supplementary Table SIV**) (32), it was hard to confirm the safety of fertility treatments in women with a family history of breast cancer. This limitation is probably due to multiple pathogenic variants of familial breast cancer. Apart from *BRCA* genes, *TP53*, *STK11*, *CDH1*, *PTEN*, *ATM*, *BARD1*, *CHEK2*, *PALB2*, *RAD51C*, *RAD51D* and other variants increase familial risk; however, more than half of the pathogenic variants in familiar breast cancer remain unclear (34, 35). The oncogenic mechanism varies among familial breast cancer cases with different genetic background. For example, *BRCA1*-related breast cancer appeared to be associated with a basal-epithelial like phenotype, *TP53* mutations are enriched in human epidermal growth factor receptor 2 (HER2) positive tumors, and *CDH1* mutations have been identified in bilateral lobular breast cancer (34–36). However, the information of subtypes of breast cancer was in lack in the eight included studies. Further studies are needed to identify the subtypes of breast cancer and specific pathogenic genes that could potentially participate in hormonal carcinogenesis.

No significant negative effect of fertility treatment was observed on women with *BRCA* mutations or separate mutations in *BRCA1* or *BRCA2*. It is speculated that *BRCA* mutation carriers are not easily affected by hormone treatment due to the clinicopathological characteristics of *BRCA*-associated cancer. Triple negative breast cancer (TNBC) is more common in *BRCA1* carriers. In most cases of *BRCA1*-associated cancer, the expressions of estrogen receptor (ER) and progesterone receptor (PR) were significantly lower (37). However, hormone receptor-positive cancers (ER-positive or PR-positive) are more frequently in *BRCA2* carriers rather than *BRCA1* carriers (38). The effect estimates with *BRCA2* carriers showed high heterogeneity (I^2 =^ 80.6%), which was probably caused by differences of sample sizes between the two studies (27, 28). In the study of Derks-Smeets (27), only three patients were diagnosed with breast cancer among the subgroup of *BRCA2* mutation carriers, while 326 *BRCA2* mutation carriers with breast cancer were included in the study of Kotsopoulos (28). The relationship between germline *BRCA* mutations and hormone-related breast cancer requires further study.

The effects of IVF, CC, and gonadotropins were evaluated separately in this study. None of the treatments was observed to increase the risk of breast cancer. However, the effects of CC remain controversial. A meta-analysis demonstrated that breast cancer risk was increased by non-IVF therapy, of which the main fertility drug was CC (33). A cohort study demonstrated that multiple CC cycles were associated with an elevated risk of breast cancer (32). However, this finding was not supported by another meta-analysis, the conclusion of which was in accordance with our study (13). The inconsistencies among the studies may have resulted from differences in the CC cycles and dosage. This implied a limitation that most studies did not explicitly describe the IVF cycles, duration, or dosage of fertility drugs. Among the included eight studies, two studies presented no association between breast cancer risk and duration or dosage of CC or gonadotropins (30, 32); two studies demonstrated that breast cancer was increased significantly by the accumulation of fertility treatment cycles (21, 31). However, the specific data of genetically susceptible women from the four studies is not available. Stratified analyses could have been used to fully evaluate the effects of specific IVF, CC, or gonadotropin regimens if data were available.

There are some limitations in this study. Eight studies included in this analysis were non-randomized observational studies, which contained three retrospective cohort studies, two prospective cohort studies, and three case-control studies. A high proportion of retrospective studies may have caused serious recall bias. Randomized trials concerned the impact of fertility treatments on breast cancer risk were not suitable based on ethical considerations. And cohort studies with large sample sizes were lacking.

Apart from study size and design, confounding factors were the main sources of high discrepancies among the included studies. Multiple confounding factors that heckled the assessment were adjusted by baseline matching, multivariable regression, or stratification. The discrepancies would be better addressed if all confounding factors such as age at menarche, parity, oral contraceptive use, subfertility, breast cancer subtypes and history of mastectomy were fully presented or adjusted in each study. Moreover, in most studies, patients with hereditary factors were not the target population, and information on genetically susceptible patients was not fully recorded or analyzed. Therefore, several studies were excluded.

The follow-up duration is important for the interpretation of the findings. A short period of follow up duration might cause a loss of record of breast cancer occurrence. Only three studies provided median or mean follow-up duration ranging from 9.69 to 30 years (21, 30, 32). Only one study described the exclusion of patients diagnosed of breast cancer in the first year after receiving fertility treatment (32). No studies reported the survival of breast cancer patients.

## Conclusion

In conclusion, the breast cancer incidence rate was not significantly increased by fertility treatments in women with a family history of breast cancer or *BRCA* mutations. Large prospective cohort studies with more detailed information such as fertility treatment regimens, history of mastectomy, and follow-up years are required. Further investigations are needed to explore subtypes of breast cancer, genetic background of hormone-related breast cancer, and the association between *BRCA* mutations and the incidence of hormone receptor-positive breast cancer.

## Data availability statement

The original contributions presented in the study are included in the article/**Supplementary Material**. Further inquiries can be directed to the corresponding authors.

## Author contributions

LW and XL designed the study. LW and XL performed database search, data extraction, and quality evaluation. XL, JY, and RP performed data analyses. XL, LW, and HZ drafted the manuscript. All authors contributed to the article and approved the submitted version.

## Acknowledgments

We would like to thank Dr Ruqiya Pervaiz for kindly providing additional information, as detailed in the manuscript. We would also like to thank the corresponding authors who replied to our request for additional information. Thank Dr. Rui Wang for providing instructional advice.

## Conflict of interest

The authors declare that the research was conducted in the absence of any commercial or financial relationships that could be construed as a potential conflict of interest.

## Publisher’s note

All claims expressed in this article are solely those of the authors and do not necessarily represent those of their affiliated organizations, or those of the publisher, the editors and the reviewers. Any product that may be evaluated in this article, or claim that may be made by its manufacturer, is not guaranteed or endorsed by the publisher.

## Supplementary material

Supplementary Figure 1 Risk bias of the included eight articles assessed by ROBINS-I. ROBINS-I, Risk Of Bias in Non-randomized Studies - of Interventions.
