# Supplementary figures and images for: Association between fertility treatments and breast cancer risk in women with a family history or BRCA mutations: a systematic review and meta-analysis
Source: Front Endocrinol (Lausanne). 2022 Sep 13;13:986477. doi: 10.3389/fendo.2022.986477 (PMC9513064; doi:10.3389/fendo.2022.986477)

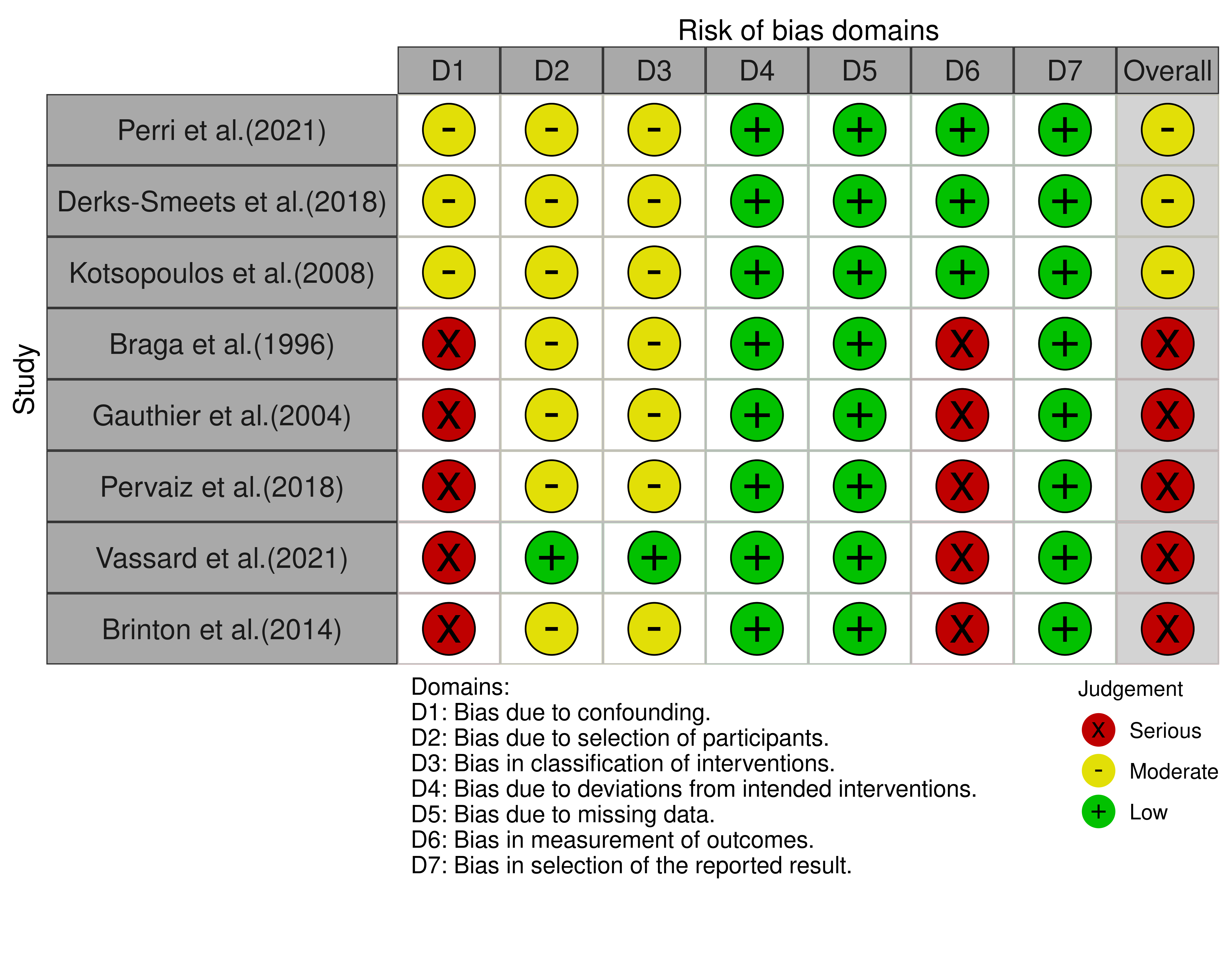

Supplement: Supplementary Figure 1 — Risk bias of the included eight articles assessed by ROBINS-I. ROBINS-I, Risk Of Bias in Non-randomized Studies - of Interventions. [file Image_1.png]
